# Apitherapy with Royal Jelly and Green Propolis EPP-AF^®^ Improves Cardiovascular Risk Markers in Patients Undergoing Hemodialysis

**DOI:** 10.3390/toxins17080369

**Published:** 2025-07-26

**Authors:** Julie Ann Kemp, Marianna Mendonça, Paloma Chrispim, Marcia Ribeiro, Isadora Britto, Karen S. Coutinho-Wolino, Marcelo Ribeiro-Alves, Lia S. Nakao, Fernanda Kussi, Eduardo B. Coelho, Andresa A. Berretta, Denise Mafra, Ludmila Cardozo

**Affiliations:** 1 Graduate Program in Nutrition Sciences, Fluminense Federal University (UFF), Niterói 24033-900, RJ, Brazil; kemp.julie@gmail.com (J.A.K.); mariemmpinho@gmail.com (M.M.); paloma_chrispim@id.uff.br (P.C.); ludmila.cardozo@gmail.com (L.C.); 2 Graduate Program in Biological Sciences–Physiology, Federal University of Rio de Janeiro (UFRJ), Rio de Janeiro 21941-941, RJ, Brazil; ribeiromarcia.trabalhos@gmail.com (M.R.); isadorabkopke@gmail.com (I.B.); karenscoutinho@gmail.com (K.S.C.-W.); 3 HIV/AIDS Clinical Research Center, Evandro Chagas National Institute of Infectious Diseases (INI/Fiocruz), Rio de Janeiro 21040-360, RJ, Brazil; mribalves@gmail.com; 4 Basic Pathology Department, Federal University of Paraná (UFPR), Curitiba 81531-980, PR, Brazil; lia.nakao@ufpr.br (L.S.N.); fernandakussi17@gmail.com (F.K.); 5 Internal Medicine Department, Ribeirão Preto Medical School, University of São Paulo (USP), São Paulo 14048-900, SP, Brazil; ebcoelho@fmrp.usp.br; 6 Research, Development & Innovation Department, Apis Flora Indl. Coml. Ltd, Ribeirão Preto 14020-670, SP, Brazil; andresa.berretta@apisflora.com.br; 7 Graduate Program in Cardiovascular Sciences, Fluminense Federal University (UFF), Niterói 24033-900, RJ, Brazil

**Keywords:** royal jelly, green propolis, cardiovascular risk markers, uremic toxins, hemodialysis

## Abstract

Background: Reducing cardiovascular risk markers is an essential target in chronic kidney disease (CKD). Thus, this study aimed to evaluate the effect of royal jelly plus green propolis supplementation on cardiovascular disease (CVD) risk factors in patients with CKD undergoing hemodialysis (HD). Methods: This randomized, double-blind, placebo-controlled trial involved HD patients allocated to receive either royal jelly plus green propolis EPP-AF^®^ (100 mg RJ + 500 mg GP) or placebo capsules daily for 2 months. Before and after the intervention period, the biochemical parameters, inflammatory cytokines, and uremic toxins were measured. Results: A total of 38 HD patients completed the 2-month supplementation study, with 19 patients in each group. After 2 months, the treated group showed a significant reduction in plasma levels of IL-6 (0.78 to 0.63 pg/mL, *p* = 0.008) and total cholesterol (138.60 to 111.85 mg/dL, *p* = 0.03), whereas no changes were observed in the placebo group. Uremic toxins did not change after intervention. Conclusion: Apitherapy with RJ + GP EPP-AF^®^ extract significantly reduced plasma IL-6 and total cholesterol in HD patients. This supplementation shows promise as a non-pharmacological strategy to reduce cardiovascular risk markers in this population.

## 1. Introduction

Chronic kidney disease (CKD) is considered a global public health concern. It is responsible for numerous premature deaths and increased morbidity. It is estimated that by 2040, it will be the 5th leading cause of years of life lost in the world [1], and cardiovascular disease (CVD) is the main cause of death among the CKD population, with 40–50% of all causes of death attributed to CVD-related [2]. Hemodialysis (HD) is the most common renal replacement therapy (RRT), accounting for around 69% of RRT in the world, and more than two-thirds of these patients are affected by CVD [3].

CVD deployment in CKD is a multifactorial cause attributed to various traditional and non-traditional risk factors, including dyslipidemia, inflammation, hypertension, oxidative stress, and uremic toxins. Regarding this, CKD patients have a specific lipid profile modification, with low high-density lipoprotein cholesterol (HDL-C) levels and hypertriglyceridemia, contributing to the CVD risk and CKD progression [4,5]. In addition, the uremic environment leads to inflammation and oxidative stress due to several factors, including the activation of the nuclear factor kappa B (NF-κB) pathway by uremic toxins, which increases the production of pro-inflammatory cytokines [6].

Managing both traditional and non-traditional risk factors is essential for reducing the incidence and progression of CVD. In this context, bioactive compounds from functional foods can aid in lowering CVD risk factors. Accordingly, apitherapy has emerged as a promising non-pharmacological approach that utilizes royal jelly and propolis, products from bees, for the prevention, treatment, and modulation of CKD and CVD progression [7].

Several studies have highlighted the cardioprotective properties of these natural compounds, primarily attributed to their antioxidant, anti-inflammatory, and immunomodulatory effects [8]. Royal jelly (RJ) and propolis are bee products. RJ is a substance produced in the hypopharyngeal glands of young worker bees, and propolis is a resinous mixture produced by bees after collecting sprouts, resins, and exudates from the *Baccharis dracundulifolia* species and combining them with their saliva and the addition of wax. Green propolis (GP) extract is obtained through the extraction process of the raw material using an alcoholic solution, following a proper procedure. GP and RJ are rich in bioactive compounds with therapeutic properties [8,9].

Several types of propolis exist, differing in color and primary bioactive compound, with green propolis (GP) being the most studied. The standardization of EPP-AF has been previously demonstrated and advantageously offers GP extracts reproducibility from batch to batch, as evidenced by the fingerprint profile and total flavonoids and phenolic [10]. Similarly, several previous studies have been conducted using in vitro and in vivo models to demonstrate the safety and efficacy of this application under various conditions [11,12]. Besides safety, this is the reason why EPP-AF^®^ was chosen for the current study.

Among all the studies that have evaluated GP properties, most are experimental models, in vitro studies, or animal models. They have demonstrated that GP has antioxidant activity and reduces inflammation and oxidative stress. All this, through modulation of the immunological system, raises anti-inflammatory cytokine (IL-10) levels and prevents the production of pro-inflammatory cytokines (IL-1β, IL-6, TNF-α, and IFN-γ) [13,14,15]. On the other hand, RJ has demonstrated antibiotic, anti-inflammatory, and antioxidant effects in in vitro and animal models [16,17,18]. This is suggested to occur due to its immunomodulatory properties, which improve the stimulation of macrophage and lymphocyte production. Moreover, RJ is proposed to modulate the signaling of some inflammatory pathways, such as NF-κB [19].

In CKD, several studies have verified the actions of GP on humans, finding that it may improve proteinuria, inflammation, and health-related quality of life (HRQoL) [20,21,22,23]. No studies were found that evaluated the effect of RJ in CKD. However, RJ was observed to be effective in reducing cisplatin nephrotoxicity in patients with cancer. In this study, RJ was found to reduce renal damage compared to the control group [24]. Thus, in the scientific literature, GP and RJ have separately demonstrated a positive effect. Furthermore, a recent literature review highlights the potential of apitherapy as a supportive strategy in drug-induced kidney injury. The underlying pathophysiological mechanisms, such as oxidative stress, inflammation, glomerular injury, increased membrane permeability, and mitochondrial dysfunction, are shared with CKD of other etiologies, suggesting broader therapeutic applicability [25]. In this study, we aimed to combine these two bee products, RJ and GP, to potentially create a compound synergy and evaluate the effectiveness of apitherapy in improving CVD markers, such as inflammatory cytokines, oxidative stress, lipid profile, and uremic toxins, in CKD patients undergoing HD.

## 2. Results

### 2.1. Participant Characteristics

**Figure 1:**
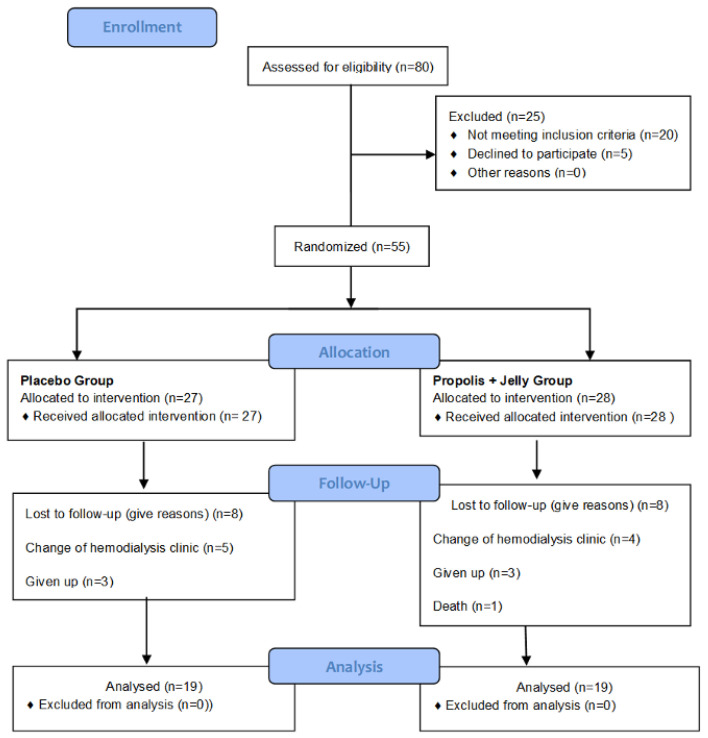
CONSORT flow diagram of the study. Dropouts were due to personal reasons unrelated to the intervention, and no adverse events were reported as reasons for discontinuation.

A total of 80 patients were evaluated to determine their eligibility, and 55 were randomized. Of these, 27 were allocated to the placebo group and 28 to the RJ + GP. Nineteen participants in each group completed the study. Eight participants in the placebo group and nine in the RJ + GP were lost to follow-up due to a change of hemodialysis clinic, withdrawal, or death (Figure 1). No intention-to-treat analysis was performed. Data were analyzed, excluding participants who did not complete the intervention period.

Table 1 presents the demographic characteristics of the study population. The baseline groups were similar, with no statistically significant differences. Briefly, we observed that overall, participants were 10.5% underweight, 36.8% eutrophic, 39.4% overweight, and 13.1% obese. However, baseline characteristics did not differ between groups. Hypertension was observed in 27 cases, with 14 in the placebo group and 13 in the RJ + GP group. Diabetes Mellitus was present in 16 cases, with 11 in the placebo group and 5 in the RJ + GP group, showing no statistically significant distribution.

The medications most used by the groups were antihypertensives (25%), oral hypoglycemic agents (44%), calcium carbonate (56%), calcitriol (18%), and erythropoietin (25%). The medications remained unchanged throughout the study.

### 2.2. Dietary Intake Profile

According to the dietary intake analysis, either group presented similar food intake at baseline (Table 2). Patients showed a low intake of energy, protein, and fiber. No significant changes were detected following the intervention.

### 2.3. Effects on Lipid Profile

Table 3 shows the standard biochemical markers. At baseline, 18.4% had a total cholesterol (TC) level lower than 100 mg/dL, 57.8% lower than 160 mg/dL, 15.7% between 161 and 190 mg/dL, and only 7.8% higher than 190 mg/dL. Moreover, a significant reduction in total cholesterol was observed in the RJ + GP group after 2 months of intervention, an effect not seen in the placebo group. In contrast, the placebo group exhibited significant increases in urea and creatinine levels, as well as a decrease in serum albumin, none of which were observed in the RJ + GP group. Moreover, at both baseline and the end of the study, we did not find any significant differences between the groups.

### 2.4. Effects on Inflammatory and Oxidative Stress Markers

**Figure 2:**
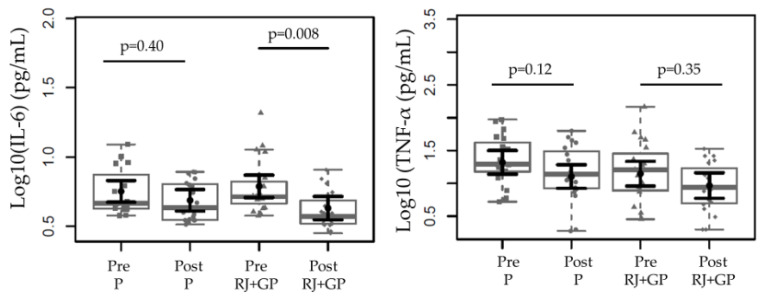
Effects of RJ + GP on inflammation (IL-6 and TNF-α) levels in both groups before and after each intervention. IL-6 was reduced in the RJ + GP group after 2 months of supplementation. Data sample distributions are displayed using box plots and strip charts in gray. In black, the central dot indicates the estimated marginal mean effect for each group derived from linear mixed-effects models. The models included the intervention group, time, their interaction, and the covariates age, sex, weight, Kt/V, and dialysis vintage as fixed effects. Participants were included as a random effect. The black horizontal lines represent the 95% confidence intervals of the predicted marginal means for each group. *p*-values were adjusted for multiple pairwise comparisons using the Tukey Honest Significant Difference (HSD) procedure. Abbreviations: IL-6, interleukin-6; TNF-α, tumor necrosis factor alpha.

RJ + GP supplementation lowered IL-6 levels from 0.78 to 0.63 pg/mL, *p* = 0.008. TNF-α levels remained relatively stable, showing no significant difference between groups. These results suggest that RJ + GP may have an anti-inflammatory effect (Figure 2).

**Figure 3:**
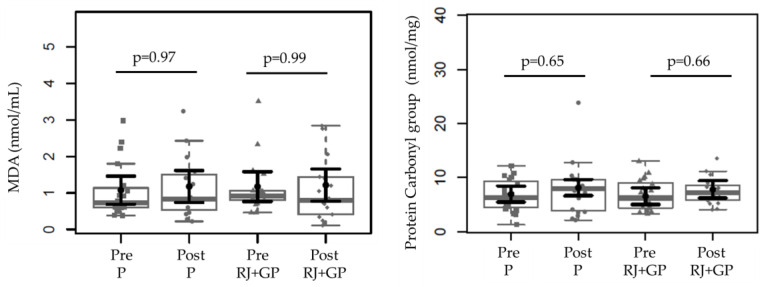
Effects of RJ + GP on oxidative stress (MDA and protein carbonyl) levels in both groups before and after each intervention. We found no differences in relative concentrations between post- and pre-supplementation in the oxidative stress parameters for placebo or RJ + GP. Data sample distributions are displayed using box plots and strip charts in gray. In black, the central dot indicates the estimated marginal mean effect for each group derived from linear mixed-effects models. The models included as fixed effects the intervention group, time, their interaction, and the covariates age, sex, weight, Kt/V, and dialysis vintage. Participants were included as a random effect. The black horizontal lines represent the 95% confidence intervals of the predicted marginal means for each group. *p*-values were adjusted for multiple pairwise comparisons using the Tukey Honest Significant Difference (HSD) procedure. Abbreviations: IL-6, interleukin-6; TNF-α, tumor necrosis factor alpha. Abbreviation: MDA, Malondialdehyde.

In Figure 3, we evaluated the effect of RJ + GP on the oxidative stress parameters. Oxidative stress was measured through plasma levels of MDA and protein carbonyls. RJ + GP supplementation after 2 months of intervention did not change these parameter levels in plasma, indicating that RJ + GP did not exert an effect on oxidative stress within this time.

### 2.5. Effects on Uremic Toxins

**Figure 4:**
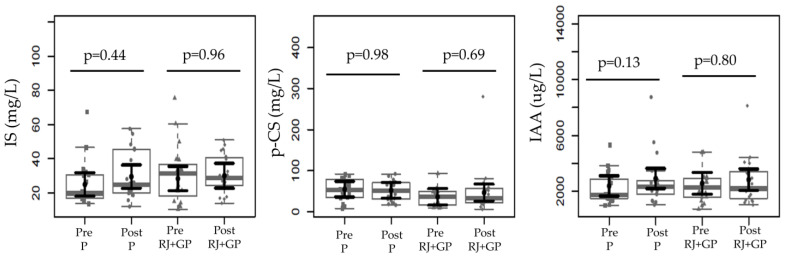
There were no changes in uremic toxins (IS, p-CS, IAA). We found no differences in relative concentrations between post- and pre-supplementation in all three uremic toxins for either the placebo or RJ + GP groups. Data sample distributions are displayed using boxplots and strip charts in gray. In black, the central dot indicates the estimated marginal mean effect for each group derived from linear mixed-effects models. The models included as fixed effects the intervention group, time, their interaction, and the covariates age, sex, weight, Kt/V, and dialysis vintage. Participants were included as a random effect. The black horizontal lines represent the 95% confidence intervals of the predicted marginal means for each group. *p*-values were adjusted for multiple pairwise comparisons using the Tukey Honest Significant Difference (HSD) procedure. Abbreviations: IL-6, interleukin-6; TNF-α, tumor necrosis factor alpha. Abbreviations: IS, indoxyl sulfate; p-CS, p-cresyl sulfate; IAA, indole-3-acetic acid.

According to data presented in Figure 4, no significant changes were observed in the concentrations of the evaluated uremic toxins, IS, p-CS, and IAA, following the 2-month supplementation period with RJ + GP. The relative plasma concentrations of IS, p-CS, and IAA remained similar before and after the intervention in both the RJ + GP and placebo groups, suggesting that RJ + GP supplementation did not affect these protein-bound uremic toxins within 2 months.

## 3. Discussion

This study evaluated the combined effects of two bee-derived products (royal jelly and green propolis) on cardiovascular risk markers in patients undergoing hemodialysis. The two-month supplementation with RJ + GP (100 mg + 400 mg/day) was designed to investigate its potential impact on inflammation, oxidative stress, and uremic toxins.

Although this supplementation brings novelty, the combination of bee products remains poorly evaluated. However, the limited literature demonstrates synergy between bee products [26]. Aboulghazi et al. (2024) combined three bee products (honey, propolis, and bee pollen) in an in vitro study to assess the potential synergy of these bee products on the antioxidant and antimicrobial effects. They demonstrated that there was a synergy between the product and improvements in antioxidant and antimicrobial activity [27]. Unfortunately, there is no study combining RJ and GP to assess their synergy in humans. However, experimental and in vitro studies combining RJ and propolis have demonstrated a significant inhibitory effect on *E. intestinalis* spore growth and improved allergic symptoms by suppressing pathways involved in the pathogenesis of allergic rhinitis [28,29].

Our intervention resulted in no significant changes in circulating uremic toxins, indicating a limited impact on gut-derived endotoxemia and toxin clearance. Although, we observed a significant reduction in IL-6 and total cholesterol levels, suggesting anti-inflammatory and lipid-lowering effects. Regarding IL-6 reduction, this parameter is very well-known to be associated with cardiovascular disease and mortality in CKD patients. As previously shown by Barreto et al. (2010), plasma interleukin-6 (IL-6) is an independent predictor of both all-cause and cardiovascular mortality in patients across different stages of chronic kidney disease [30]. In addition, Istanbuly et al. (2023) confirmed a negative correlation between circulating IL-6 concentrations and survival in patients undergoing dialysis in a systematic review and meta-analysis study [31]. Given this, the significant reduction in IL-6 observed in our study suggests that supplementation with royal jelly plus green propolis could have important clinical implications by improving and reducing CVD risk in CKD patients.

Although bee products have been used in human health for centuries, only a limited number of studies have evaluated the effects of green propolis in patients with CKD, particularly those undergoing hemodialysis. To date, no clinical trials have assessed the isolated use of royal jelly in this population. Our previous studies with HD patients have reported that GP (400 mg/day) can reduce TNF-α and exhibit a lowering tendency of the macrophage inflammatory protein-1β (MIP-1β). At the same time, there was a significant augmentation in the nuclear factor erythroid 2-related factor 2 (Nrf2) expression, which is associated with antioxidant response [21,22]. Moreover, Silveira et al. (2019) also found that 500 mg/day of GP reduced proteinuria in both non-dialysis CKD patients over 12 months [20].

Regarding this, a recent systematic review that included both human and animal studies suggested that propolis supplementation could have potentially beneficial effects on mitigating renal function in CKD [32]. Thus, our current results from this study reinforce those of previous studies, suggesting that GP has an anti-inflammatory effect in HD patients. A possible molecular pathway for this GP effect was explored by Asgharpour et al. (2019) [33]. The authors, through an in vitro study using murine macrophage (RAW 264.7) cells, found that the propolis extract inhibited the proliferation of these cells, which is a cell lineage of macrophages associated with inflammation. Moreover, the authors also found a reduction in reactive oxygen species (ROS) and nitric oxide (NO) production, as well as a decrease in the expression of cyclooxygenase-2 (Cox-2), IL-1β, and IL-6. This study, using biological evidence, demonstrated that propolis extract decreases the production of ROS and NO, thereby reducing the production of pro-inflammatory cytokines [33]. Despite this, propolis has been shown to inhibit and downregulate some pro-inflammatory pathways, including TLR4, NLRP inflammasomes, and NF-κB. Hence, decreasing the production of IL-1β, IL-6, IFN-γ, and TNF-α [34].

No studies have been conducted in patients with CKD using RJ. Furthermore, most studies using RJ are based on experimental animal models or cell culture [35]. Recently, Ogawa et al. evaluated the use of sebacic acid (SA), which is a fatty acid derived from royal jelly, for its modulatory effects on inflammatory cytokine expression in LPS-stimulated THP-1-derived macrophage-like cells. SA significantly downregulated the mRNA expression of IL-6 induced by LPS stimulation, while the expression levels of TNF-α and interleukin-1 beta (IL-1β) remained unaffected [36]. Furthermore, celecoxib-induced rats showed increased oxidative stress, and co-supplementation with 300 mg/kg/day RJ attenuated the increases in malondialdehyde (MDA) and the reduction in superoxide dismutase (SOD) levels [37]. According to these studies, RJ has distinct molecular pathways that regulate inflammation, oxidative stress, the immune system, and other processes [38,39,40]. Despite the number of clinical trials using RJ, it is suggested that RJ may improve serum glucose in people with type 2 diabetes and decrease total cholesterol and LDL-C in hypercholesterolemic adults [41,42]. In our study, we also observed the reduction in total cholesterol in the RJ + GP group, which could be attributed to the RJ.

Kamakura et al. (2006) [43] found that mice fed RJ had reduced gene expression of squalene epoxidase (SQLE), an enzyme responsible for cholesterol synthesis. Additionally, they observed a reduction in the gene expression of the sterol regulatory element-binding protein (SREBP-1), a transcription factor that may regulate SQLE expression. Also, the RJ increased the gene expression of low-density lipoprotein receptor (LDLR). Thus, the authors in this significant study demonstrated that RJ, through SQLE and LDLR regulation, has a hypocholesterolemic action [43].

Concerning uremic toxins, CKD patients are prone to having high values of uremic toxins due to kidney filtration decline and gut dysbiosis. Currently, some studies have demonstrated the effect of GP or RJ on gut microbiota modulation, gut permeability, and uremic toxins. Chang et al. (2020) observed an improvement in tubulointerstitial fibrosis (TIF) in an animal model treated with propolis extract, which affected the fibrotic epithelial–mesenchymal transition and the transforming growth factor beta (TGF-β) pathway, thereby enhancing the elimination of uremic toxins through filtration [44]. However, Fonseca et al. (2024), who administered 400 mg/day of green propolis extract for 2 months to HD patients, did not find any differences in gut microbiota composition or levels of uremic toxins [45]. Still, no studies exist using RJ to assess its effect on uremic toxins. Chi et al. (2021) [46] administered RJ in the food of healthy mice, which promoted modifications in network interactions within the gut microbiome and between the host. Moreover, they observed an increase in serum IL-10 levels, a pro-inflammatory cytokine [46]. Given the idea that GP and RJ could act on the gut microbiota and possibly reduce uremic toxins, we tested the RJ + GP supplementation on uremic toxins (IS, p-CS, and IAA) in HD patients, but we did not find any alteration.

Lastly, this study has some limitations. First, the small sample size may have limited our ability to detect differences in certain variables of interest. Second, the intervention duration may have been insufficient, as many similar studies typically last at least 12 weeks, which could be necessary to observe changes in uremic toxins and oxidative stress markers. Third, the dosage used might not have been sufficient to produce measurable effects on these specific endpoints. It is essential to note that there are no studies evaluating the combination of RJ and GP; therefore, the doses used in this study were based on previous studies evaluating each compound separately, with a primary focus on patient safety. Additionally, the clearance effects of hemodialysis masked potential changes in gut-derived uremic toxins. Moreover, due to unforeseen circumstances during the study execution, including technical and logistical limitations, it was not possible to analyze the primary outcomes (NF-κB and gut microbiota composition) registered in ClinicalTrials.gov. Finally, a crossover design could have increased statistical power and minimized intra-individual variability.

However, our study also has notable strengths. It was a well-conducted and controlled clinical trial, and to our knowledge, the first to evaluate the combination of RJ and GP on cardiovascular risk markers in patients undergoing hemodialysis. Furthermore, RJ and GP are natural products with a good safety profile, making them attractive as complementary therapies in clinical practice.

## 4. Conclusions

In conclusion, supplementation with RJ + GP was effective in reducing IL-6 and total cholesterol, suggesting potential cardiovascular benefits for HD patients. Given that CVD is the leading cause of death in this population, interventions that can improve its risk markers are of particular importance. However, RJ + GP did not affect uremic toxins, indicating that its effects may be limited to inflammatory and lipid parameters. Despite this, RJ + GP could be a promising nutritional strategy to improve CVD markers in HD patients. Further studies are needed to confirm these results in larger and more diverse populations. Additionally, future research should investigate the dosage and potential pharmacokinetic interactions.

## 5. Materials and Methods

### 5.1. Study Design and Population

This is a longitudinal, double-blind, randomized, placebo-controlled clinical trial involving patients with chronic kidney disease (CKD) undergoing hemodialysis at Hospital São José do Avaí, located in Itaperuna, Rio de Janeiro, Brazil. For sample size calculation, we assumed that TNF-alpha concentration would decrease by 1.0 pg/mL after the intervention, using a within-patient deviation of 2.3. Considering a Type I error of *p* < 0.05 and a test power of 80%, we estimated that 25 patients per group would be required (Kohn & Senyak).

The study was conducted after obtaining informed consent, in compliance with the guidelines of the local Research Ethics Committees and the requirements established by Resolution No. 466, 12 December 2012 (National Health Council). These regulations are based on the Declaration of Helsinki and the World Medical Association’s ethical principles for research involving human subjects. This study was approved by the Research Ethics Committee of the Faculty of Medicine of the Antônio Pedro University Hospital, Fluminense Federal University (UFF), under protocol number CAAE: 69096023.5.0000.5243. The trial was registered at ClinicalTrials.gov (Identifier: NCT06288204).

Demographic and clinical data were gathered through medical record analysis and patient interviews conducted during consultations or follow-ups. Biological samples were obtained before an HD session. The inclusion criteria for the study were men and women aged 18 to 75 years who underwent hemodialysis (HD) for at least 6 months, with an arteriovenous fistula (AVF) as the vascular access. They followed an individualized dietary prescription with a caloric intake of 25 to 35 kcal/kg/day and protein intake of 1.0–1.2 g/kg/day, as recommended by the NKF-KDOQI (2020) guidelines. The exclusion criteria included patients with autoimmune or infectious diseases, pregnant and lactating women, patients diagnosed with cancer or AIDS, patients using catabolic drugs, patients who had taken antibiotics within the last 3 months, patients using prebiotics, probiotics, or symbiotics, individuals with habitual consumption of propolis or royal jelly, and patients with allergies to corn starch or bee stings, due to the composition of the intervention capsules.

The randomization was computer-generated in a 1:1 ratio by an outside researcher to maintain the study’s double-blind nature. The participants were allocated into one of two groups: the Royal jelly + green propolis group standardized extract (EPP-AF^®^, Apis Flora Industrial e Comercial Ltda, Ribeirão Preto, SP, Brazil), called RJ + GP, or the placebo group. Participants received four capsules daily, providing a total of 100 mg of royal jelly and 500 mg of propolis per day for 2 months. The placebo group received the same number of capsules, at the exact times, containing only the vehicle of the active formulation. Apis Flora Industrial e Comercial Ltd., Ribeirão Preto, SP, Brazil, prepared these capsules. Anthropometric assessments, dietary intake evaluations, and blood sample collections were performed at baseline and during follow-up visits.

During the intervention period, participants were monitored at every hemodialysis session and contacted by phone to encourage adherence to the treatment and to identify potential adverse effects or intolerances. At the end of the study, the remaining capsules were counted to assess compliance. This study was analyzed on a per-protocol basis, with data from participants who adhered to the intervention protocol and completed both baseline and follow-up assessments included in the final analysis.

#### 5.1.1. Anthropometric Analysis

Nutritional status assessment included measurements of body weight and height. Anthropometric data were obtained using the following equipment: a Welmy electronic scale (with a 200 kg capacity) and a stadiometer. Nutritional status was evaluated using Body Mass Index (BMI), calculated as weight divided by height squared, and classified according to the World Health Organization (WHO, 1998) criteria.

#### 5.1.2. Food Intake

Dietary intake was assessed at baseline and after two months of intervention using a three-day 24 h dietary recall, two days from the week, and one from the weekend. The analysis of energy and macronutrient intake was performed using the DietBox^®^ software (version 7.0.12). Energy and macronutrient intake were calculated for each day, and then the median value for each parameter was determined.

#### 5.1.3. Biochemical Analysis

Blood samples were collected in EDTA tubes (BD Vacutainer® K2EDTA, Becton Dickinson, Franklin Lakes, NJ, USA) and BD™ Vacutainer™ (Becton Dickinson, Franklin Lakes, NJ, USA) Serum Tubes after a 12 h fasting period. Whole blood was centrifuged at 3500 rpm for 15 min at 4 °C to obtain plasma and serum, which were then stored at −80 °C until further analysis at the Clinical Research Unit (UPC) of Antonio Pedro’s University Hospital (HUAP).

Routine biochemical parameters, including urea, creatinine, albumin, lipid profile, calcium, potassium, and phosphorus, were determined using a commercial Bioclin kit and analyzed using a BioClin^®^ (Quibasa Química Básica Ltda., Belo Horizonte, Brazil) automated analyzer following the company protocol for each at the LABNE laboratory in the Nutrition Institute of Federal Fluminense University (UFF). The low-density lipoprotein was assessed using the following formula: LDL = (CT-HDL) − (TG/5) [47].

### 5.2. Uremic Toxin Analysis

Plasma levels of indoxyl sulfate (IS), p-cresyl sulfate (p-CS), and indole-3-acetic acid (IAA) were analyzed using reverse-phase liquid chromatography (RP-LC) in collaboration with the Federal University of Paraná (UFPR). Chromatographic determinations were performed with a Shimadzu Prominence system equipped with a quaternary pump (Shimadzu LC-20AD, Shimadzu Corporation, Kyoto, Japan), controlled by the LC Solution software(version 1.25 SP5), a fluorescence detector (Shimadzu RF-20A), and an autosampler (Shimadzu SIL10AF, Shimadzu Corporation, Kyoto, Japan). Separation was achieved using a 150 × 4.6 mm, 5 µm C8 Luna column (Phenomenex Inc., Torrance, CA, USA), eluted with 50 mM ammonium formate, pH 3.0, and methanol, with the proportion of methanol increasing from 35% to 70% at a flow rate of 0.7 mL/min. During the run, the fluorescence wavelengths varied: λexc = 280 nm and λem = 383 nm for IS and IAA [48] and λexc = 265 nm and λem = 290 nm for p-CS [49]. 4-Ethylphenol was used as an internal standard.

### 5.3. Determination of Inflammatory Cytokines IL-6 and TNF-α

The concentrations of the inflammatory cytokines IL-6 and TNF-α were measured using commercial ELISA kits (R&D Systems^®^). Quantitative sandwich ELISA kits (PeproTech Inc., Cranbury, NJ, USA) were employed to detect TNF-α and IL-6 within a range of 31–2000 pg/mL, following a standard protocol. Inflammatory cytokine levels (IL-6 and TNF-α) in plasma were measured using Peprotech^®^ ELISA kits (IL-6 ELISA kit, no. 900-T16, Cranbury, NJ, USA, and TNF-α ELISA kit, no. 900-T25, Cranbury, NJ, USA) following the manufacturer’s protocol. Human capture antibodies for TNF-α and IL-6 were diluted in PBS to a concentration of 1.0 μg/mL for TNF-α and 0.50 μg/mL for IL-6, and used to coat 96-well Maxisorp microplates (Nunc, Thermo Fisher Scientific, Leicestershire, UK) overnight at room temperature. After the wells were aspirated and washed with wash buffer, the block buffer was added for incubating for 1 h and 30 min. A new wash was performed, and then the standard curve and samples were added to the plate and incubated at room temperature for two hours. Later, the plate was aspirated, washed, and detection antibody was added at a concentration of 0.15 μg/mL for TNF-α and 0.1 μg/mL for IL-6, and incubated at room temperature for two hours. After the plate was aspirated and washed, a streptavidin conjugate was added to a concentration of 0.05 μg/mL and incubated for 30 min. Finally, the plate was again aspirated and washed, and TMB liquid substrate was added for color development and subsequent reading at 450 nm. The results were log-transformed.

### 5.4. Determination of Lipid Peroxidation

Lipid peroxidation was used as a marker of oxidative stress, assessed by thiobarbituric acid-reactive substances (TBARS) using the modified Ohkawa method [50]. Samples were diluted with thiobarbituric acid (0.6% *w*/*v*), SDS (8.1% *w*/*v*), and phosphoric acid (1% *w*/*v*), and then heated at 95 °C for 60 min. The microtubes were centrifuged at 4000 rpm for 20 min at 20 °C. The supernatant was then collected, and the absorbance was measured at 532 nm using a Synergy H1M microplate reader (Biotek Instruments, Winooski, VT, USA). Plasma TBARS levels were expressed as nanomoles per milliliter.

### 5.5. Determination of Plasma Protein Carbonyl Content

Protein carbonylation in plasma was assessed using a method adapted from Levine et al. (1990) [51]. In total, 50 μL of plasma samples were mixed with 500 μL of 10% trichloroacetic acid and then centrifuged for 2 min at 2.000× *g*. After the supernatant was aspirated, 500 μL of 2,4-dinitrophenylhydrazine (DNPH) was added to the test samples, and 500 μL of 2 M hydrochloric acid was added to the control samples. After being incubated for 15 min at room temperature, 500 μL of 10% trichloroacetic acid was added to all samples, and then they were centrifuged for 2 min at 2000× *g*. Later, the supernatant was aspirated, and the samples were washed twice with ethanol and ethyl acetate. The samples were then centrifuged again for 2 min at 2000 × *g*. The supernatant was discarded, and the samples were resuspended with 400 μL of guanidine. Carbonyl content was determined spectrophotometrically at 370 nm using a PowerWave XS microplate reader (Biotek^®^ Instruments, Winooski, VT, USA). Results were expressed as nanomoles of carbonyl per milligram of protein. Protein concentration was determined by the Bradford method based on the binding of Coomassie Brilliant Blue dye.

#### Statistical Analysis

Data were expressed as median (interquartile range). Baseline demographic and clinical variables, expressed as continuous numerical data, were compared using the non-parametric Mann–Whitney U test. Categorical variables were analyzed using Chi-squared tests to compare relative frequencies.

Linear mixed-effects models were used to evaluate time-by-intervention interactions. Multiple linear fixed-effects models were used to assess differences between the baseline and two-month time points, adjusting for potential confounders (age, sex, weight, Kt/V, and time on hemodialysis). Graphical representations display the estimated average marginal effects, along with their corresponding 95% confidence intervals. The Tukey Honest Significant Difference (HSD) method was applied to adjust *p*-values for multiple comparisons. Pearson’s adjusted correlation coefficient (ρ) was used to examine correlations between variables. Statistical significance was defined as *p* < 0.05. All analyses were performed using R, version 4.1.3.

## Figures and Tables

**Table 1:** General characteristics in the RJ + GP and the placebo group at the baseline.

Parameters	Overall	RJ + GP Group (N = 19)	Placebo Group (N = 19)	*p*-Value
Sex (Female/Male)	12/26	7/12	5/14	0.72
Age (years)	59.5 (17.0)	59.0 (18.5)	60.0 (16.5)	0.91
BMI (Kg/m^2^)	25.7 (5.8)	24.2 (4.8)	25.9 (10.7)	0.45
Kt/V	2.4 (0.5)	2.3 (0.6)	2.4 (0.7)	0.18
HD vintage (months)	57.0 (62.2)	48.0 (41.5)	60.0 (69.0)	0.18

Data are presented as either absolute (relative) frequencies or median (IQR). *p*-values were estimated by either chi-squared or non-parametric Mann–Whitney U tests. Abbreviations: BMI: Body Mass Index; HD: Hemodialysis; Kt/V: Kinetic index of dialysis adequacy.

**Table 2:** Dietary intake analysis of the RJ + GP group and the placebo group at baseline.

Dietary Variables	RJ + GP Group (N = 19)	Placebo Group (N = 19)	*p*-Value
Energy (kcal/day)	1576.8 (1314.4–1839.1)	1202.4 (940.9–1463.3)	0.54
Carbohydrates (g/day)	187.0 (153.8–220.3)	147.4 (114.3–180.6)	0.28
Lipids (g/day)	55.0 (44.2–65.8)	40.3 (29.5–51.1)	0.19
Protein (g/kg/day)	1.0 (0.9–1.2)	0.9 (0.8–1.1)	0.88
Potassium (mg/day)	1789.6 (1528.5–2050.7)	1398.3 (1138–1658.6)	0.12
Phosphorus (mg/day)	919.3 (767.7–1071.0)	680.7 (529.6–831.9)	0.09
Sodium (mg/day)	1785.3 (1393.3–2177.4)	1324.5 (933.7–1715.3)	0.30
Fibers (g/day)	16.1 (12.0–20.2)	16.6 (12.6–20.7)	0.99

Data are presented as median (Q1–Q3). *p*-values were estimated by non-parametric Mann–Whitney U tests.

**Table 3:** Biochemical parameters of the RJ + GP group and the placebo group.

Variables	RJ + GP (N = 19)		*p*-Value	Placebo (N = 19)		*p*-Value
	Baseline	Post		Baseline	Post	
Glucose (mg/dL)	106.2 (88.2–124.2)	106.0 (87.6–124.5)	0.99	106.7 (89.9–123.5)	118.7 (101.9–135.4)	0.54
Phosphorus (mg/dL)	5.0 (4.1–5.9)	4.6 (3.7–5.5)	0.74	4.4 (3.6–5.3)	4.5 (3.7–5.4)	0.99
Potassium (mg/dL)	5.5 (5.0–6.0)	5.4 (4.8–5.9)	0.98	5.1 (4.6–5.6)	5.5 (5.0–6.0)	0.55
Urea (mg/dL)	126.5 (108.1–144.8)	134.2 (115.2–153.1)	0.91	96.8 (79.6–114.0)	132.7 (115.6–149.8)	**0.007**
Creatinine (mg/dL)	11.2 (9.4–12.9)	11.7 (9.9–13.5)	0.88	8.4 (6.7–10.1)	10.9 (9.2–12.6)	**0.001**
Albumin (mg/dL)	4.1 (3.8–4.5)	3.7 (3.4–4.1)	0.25	4.3 (4.0–4.6)	3.7 (3.4–4.0)	**0.03**
Total cholesterol (mg/dL)	140.5 (121.8–159.1)	113.7 (94.8–132.6)	**0.03**	136.2 (118.8–153.6)	117.1 (99.8–134.5)	0.10
Triglycerides (mg/dL)	171.7 (116.4–227.1)	138.4 (83.2–193.6)	0.33	156.9 (104.8–209.0)	141.2 (89.2–193.2)	0.77
HDL-C (mg/dL)	41.9 (36.3–47.6)	37.4 (31.8–43.1)	0.09	38.5 (33.1–43.8)	41.9 (36.3–47.6)	0.51
LDL-C (mg/dL)	63.2 (49.0–77.4)	50.0 (34.9–65.0)	0.28	66.6 (53.0–80.3)	52.9 (39.3–66.5)	0.14

Data are presented as median (Q1–Q3). Linear regression analysis, adjusted for age, sex, and body mass index (BMI), was performed to compare the four groups. *p* values < 0.05 were considered statistically significant.

## Data Availability

The original contributions presented in this study are included in the article. Further inquiries can be directed to the corresponding author(s).

## Abbreviations

CKD	Chronic kidney disease
CVD	Cardiovascular disease
HDL-C	High-density lipoprotein cholesterol
NF-kB	Nuclear factor kB
RJ	Royal jelly
GP	Green propolis
P	Propolis
IL-10	Interleukin 10
IL-1β	Interleukin 1 beta
IL-6	Interleukin 6
TNF-α	Tumor Necrosis Factor alpha
IFN-γ	Interferon gamma
HRQoL	Health-related quality of life
DM	Diabetes Mellitus
BMI	Body mass index
Kt/V	Kinetic index of dialysis adequacy
HD	Hemodialysis
LDL-C	Low-density lipoprotein cholesterol
MDA	Malondialdehyde
IS	Indoxyl Sulfate
p-CS	P cresyl sulfate
IAA	Indole-3-acetic acid
HDS	Tukey Honest Significant Difference
Nrf2	Nuclear factor erythroid 2-related factor 2
MIP-1β	Macrophage inflammatory protein-1β
ROS	Reactive oxygen species
NO	Nitric oxide
Cox-2	Cyclooxygenase-2
mRNA	Messenger ribonucleic acid
LPS	Lipopolysaccharide
SA	Sebacic acid
SOD	Superoxide Dismutase
SQLE	Squalene epoxidase
SREBP-1	Sterol regulatory element-binding protein
LDLR	Low-density lipoprotein receptor
TIF	Tubulointerstitial fibrosis
TGF-β	Transforming growth factor beta
TLR4	Toll-like receptor 4
